# IL-18 polymorphisms contribute to hepatitis B virus-related cirrhosis and hepatocellular carcinoma susceptibility in Chinese population: a case-control study

**DOI:** 10.18632/oncotarget.18531

**Published:** 2017-06-17

**Authors:** Zhi-Jun Dai, Xing-Han Liu, Meng Wang, Yan Guo, Wenge Zhu, Xiao Li, Shuai Lin, Tian Tian, Kang Liu, Yi Zheng, Peng Xu, Tianbo Jin, Xiaopeng Li

**Affiliations:** ^1^ Department of Oncology, The Second Affiliated Hospital of Xi’an Jiaotong University, Xi’an, China; ^2^ School of Life Science and Technology, Xi’an Jiaotong University, Xi’an, China; ^3^ Department of Biochemistry and Molecular Medicine, The George Washington University Medical School, Washington, DC, USA; ^4^ Department of Hepatobiliary Surgery, Xijing Hospital, Fourth Military Medical University, Xi'an, China; ^5^ National Engineering Research Center for Miniaturized Detection Systems, School of Life Sciences, Northwest University, Xi’an, China; ^6^ Department of Ultrasound, The Second Affiliated Hospital of Xi’an Jiaotong University, Xi’an, China

**Keywords:** IL-18, HBV, susceptibility

## Abstract

IL-18 polymorphisms influence the transcriptional activity of the IL-18 gene and associated with various diseases. However, their relationships with hepatitis B virus-related liver diseases had not reached a consensus. So we conducted this case-control study with a view to clarifying the association. We included four groups: healthy controls, chronic hepatitis B virus (CHB) carriers, liver cirrhosis (LC) and hepatocellular carcinoma (HCC) groups with each group of 250 persons. Odd ratios (ORs) and 95% confidence intervals (95%CIs) with or without adjustment were calculated. Haplotype analysis was also performed. The results showed people carrying rs187238 CG genotype had a lower risk of LC (CG vs. CC: OR = 0.59, 95%CI = 0.38–0.91, *P* = 0.02), while GG genotype carriers had a higher risk of HCC (GG vs. CC+CG: OR = 4.73, 95%CI = 1.01–22.1, *P* = 0.03) than those with CC and CG genotypes in healthy group. Rs187238 GG genotype increased the risk from CHB to LC status (GG vs. CC: OR = 4.81, 95%CI = 1.03–22.6; GG vs. CC+CG: OR = 4.73, 95%CI = 1.01–22.1), meanwhile the trend also existed by controlling confounding factors (GG vs. CC: OR = 6.25, 95%CI = 1.09–35.8; GG vs. CC+CG: OR = 5.91, 95%CI = 1.04–33.7). Haplotype Crs187238Trs1946518 moderately decreased the risk of CHB carriers developing into HCC (OR = 0.69, 95%CI = 0.50–0.96, *P* = 0.03) after adjustment. In conclusion, IL-18 rs187238 GG genotype may increase the risk of HCC in healthy population and the risk of LC in CHB carriers.

## INTRODUCTION

From 1990 to 2013, ranking of viral hepatitis on leading death causes worldwide rose from the tenth to seventh [1]. Despite the availability of an effective vaccine and antiviral approaches, more than 250 million people suffer from chronic hepatitis B (CHB) infection and nearly one million deaths occur each year owing to complications worldwide [2, 3]. Genomic mutations including drug-resistant mutants and vaccine-escape mutants (G145R, G145A, F134L and others) hinder the treatment and prevention of hepatitis B virus (HBV) infection [2]. Prospective studies revealed chronically infected HBV patients had an up to 100-fold increased risk for progressing to hepatocellular carcinoma (HCC) [4]. Genomic mutations were significantly involved in the development of HBV-related HCC. P203Q and S210R which are mutations in HBsAg C-terminus hamper HBsAg-secretion and correlate with increased cellular proliferation and HBV-induced HCC [5]. A meta-analysis performed by Tian T et al. indicated that miR-146a C>G increased HBV-related HCC risk while miR-196a-2 C>T decreased the risk of HBV-related HCC, especially in the Chinese population [6]. A retrospective study found the AG genotype and G allele for A3G rs8177832 polymorphism related to a decreased risk of CHB and HBV-related HCC and TT genotype of rs2011861 polymorphism confer to an increased risk of HBV-related HCC [7].

Interleukin-18 (IL-18), firstly described as interferon (IFN)-gamma inducing factor, is one of the members of the IL-1 cytokine superfamily [8, 9]. IL-18 modulates the Th1 response together with IL-12 to produce IFN-gamma [10]. It has been shown that IL-18 can also influence Th2 response in synergy with other Th2-stimulating factors [11]. Thus, IL-18 is capable of increasing the activity of both Th1-type and Th2-type CD4^+^ T cells depending on its cytokine milieu [12]. CD4^+^ T cells can contribute directly to disease pathogenesis and inhibit viral replication during HBV infection [13]. Cytotoxic T lymphocytes can directly recognize and kill infected hepatocytes [14]. IL-18 plays a part in the clearance of viruses, partly by the induction of cytotoxic T cells [15]. All the findings suggest IL-18 may be associated with HBV and HCC. Human IL-18 gene is located on chromosome 11q22.2–22.3 and contains six exons. Within the promoter region of the IL-18 gene, two polymorphisms (rs187238 and rs1946518) have been identified, which influence the transcriptional activity of the IL-18 gene [16]. Previous studies demonstrated an significant association between rs1946518 polymorphism and various diseases risk, such as asthma, Crohn's disease and breast cancer [17–19]. Rs1946518 polymorphism was also suggested to be associated with hepatitis C virus (HCV) infections [20]. Hass SL et al. found though different genotypes of the two SNPs distributed equally in HCV patients and health controls, they were related to treatment response in hepatitis C patients [21]. Li Y et al. found no association between the two SNPs and HBV recurrence after liver transplantation in Chinese Han people [22].

Several researches have studied the impact of the two polymorphisms on HBV-induced liver diseases, but no consistent conclusions had been achieved [23–26]. The purpose of this study was to assess the relationship of rs187238 and rs1946518 polymorphisms and the risk of HBV-liver cirrhosis (LC) and HBV-related HCC in the northwestern Chinese population.

## RESULTS

### Characteristics of the study population

The included subjects were equally categorized into four groups with 250 participants, healthy controls, CHB, CHB-positive LC and CHB-positive HCC (Table 1). The average (±standard deviation) of the four parts were 55.71 ± 9.17, 54.17 ± 10.37, 53.12 ± 10.58 and 54.42 ± 12.00, respectively. Males occupied 80.4%, 72.8%, 70.8% and 77.6% in the healthy group, CHB carriers, LC and HCC patients, respectively. The four groups did differ significantly in alcohol history (*P* = 0.002) and diabetes history (*P* = 0.021), while they had no meaningful difference in smoking history (*P* = 0.100) and family history (*P* = 0.647). Furthermore, patients carrying CHB related liver diseases had higher laboratory parameters than healthy controls, including T-Bil, ALT and AST levels (*P* <0.001), which are evidences of liver injury [27].

**Table 1:** Characteristics of included subjects

Characteristics	Healthy controls	CHB	CHB-related LC	CHB-related HCC	*P*
Total number	250	250	250	250	
Age (mean ± SD)	55.71 ± 9.17	54.17 ± 10.37	53.12 ± 10.58	54.47 ± 12.00	0.056
Gender					
Male	201	182	177	194	0.051
Female	49	68	73	56	
Alcohol history					
yes	22	53	39	42	**0.002**
no	228	197	211	208	
Smoking history					
yes	145	137	118	131	0.100
no	105	113	132	119	
Diabetes history					
Yes	22	30	39	44	**0.021**
no	228	220	211	206	
Family history					
Yes	8	7	12	10	0.647
No	242	243	238	240	
Laboratory parameters					
T-Bil level (umol/L)	8.49 ± 4.09	14.35 ± 5.29	39.22 ± 11.58	36.76 ± 10.11	**< 0.001**
ALT (U/L)	15.36 ± 5.27	65.33 ± 13.40	63.84 ± 21.26	71.03 ± 16.50	**< 0.001**
AST (U/L)	12.35 ± 4.05	49.62 ± 15.16	47.99 ± 24.83	58.21 ± 17.55	**< 0.001**
AFP (ng/ml)	No data	7.94 ± 3.31	43.43 ± 29.46	1629.59 ± 625.28	**< 0.001**
Number of patients with AFP ≤400 ng/ml	−	−	−	156	

CHB, chronic hepatitis B; LC, liver cirrhosis; HCC, hepatocellular carcinoma; AFP, alpha-fetoprotein.

The alleles and genotype distributions of IL-18 polymorphisms were shown in Table 2. Genotype frequencies of the two polymorphisms in control group were consistent with HWE (Table 3, rs187238: *P* = 0.142; rs1946518: *P* = 0.90). A LC patient and five HCC patients were failed to detect 187328 genotype, and three patients with HCC was failed to detect rs1946518 polymorphism. For rs187238 polymorphism, as displayed in Table 2, healthy controls with CC, CG and GG genotypes were 183, 65 and 2; CHB carriers were 200, 48, and 2; LC patients were 202, 42 and 5, and HCC subjects were 187, 49 and 9, respectively. For rs1946518 polymorphism, GG, GT and TT genotype accounts for 25.6%, 49.6% and 24.8% in disease free controls, 24.4%, 53.6% and 22% in CHB carriers, 28.8%, 47.2% and 24% in LC patients, and 26.8%, 47.2% and 24.8% in HCC patients, respectively (Table 2).

**Table 2:** Allele and genotype distributions of rs187238 and rs1946518 polymorphisms in healthy controls, CHB, LC and HCC patients

Model		Control (*n*, %)	CHB (*n*, %)	LC (*n*, %)	HCC (*n*, %)
rs187238					
Allele	C	431 (86.2%)	448 (89.6%)	446 (89.6%)	423 (86.3%)
	G	69 (13.8%)	52 (10.4%)	52 (10.4%)	67 (13.7%)
Codominant model	CC	183 (73.2%)	200 (80%)	202 (81.1%)	187 (76.3%)
	CG	65 (26%)	48 (19.2%)	42 (16.9%)	49 (20%)
	GG	2 (0.8%)	2 (0.8%)	5 (2%)	9 (3.7%)
Dominant model	CC	183 (73.2%)	200 (80%)	202 (81.1%)	187 (76.3%)
	CG+GG	67 (26.8%)	50 (20%)	47 (18.9%)	58 (23.7%)
Recessive model	CC+CG	248 (99.2%)	248(99.2%)	244 (98%)	236 (96.3%)
	GG	2 (0.8%)	2 (0.8%)	5 (2%)	9 (3.7%)
rs1946518					
Allele	G	252 (50.4%)	256 (51.2%)	262 (52.4%)	252 (51%)
	T	248 (49.6%)	244 (48.8%)	238 (47.6%)	242 (49%)
Codominant model	GG	64 (25.6%)	61 (24.4%)	72 (28.8%)	67 (27.1%)
	GT	124 (49.6%)	134 (53.6%)	118 (47.2%)	118 (47.8%)
	TT	62 (24.8%)	55 (22%)	60 (24%)	62 (25.1%)
Dominant model	GG	64 (25.6%)	61 (24.4)	72 (28.8%)	67 (27.1%)
	GT+TT	186 (74.4%)	189 (75.6%)	178 (71.2%)	180 (72.9%)
Recessive model	GG+GT	188 (75.2%)	195 (78%)	190 (76%)	185 (74.9%)
	TT	62 (24.8%)	55 (22%)	60 (24%)	62 (25.1%)
Haplotype	GT	13.80%	10.40%	10.44%	13.52%
	CT	35.80%	38.40%	36.95%	35.66%
	CG	50.40%	51.20%	52.61%	50.82%

HWE = hardy-weinberg equilibrium, CHB = chronic hepatitis B, LC = liver cirrhosis, HCC = hepatocellular carcinoma, GT, G_rs187238_T_rs1946518_; CT, C_rs187238_T_rs1946518_; CG, C_rs187238_G_rs1946518_.

**Table 3:** Hardy-Weinberg equilibrium test for the rs187238 and rs1946518 polymorphisms in the control group

	Observed frequency	Expected frequency	*P*
Rs187238			
CC	183	185.761	0.142
CG	65	59.478	
GG	2	4.761	
Rs1946518			
GG	64	63.504	0.900
GT	124	124.992	
TT	62	61.504	

### Association between IL-18 polymorphisms and the risk of LC

Compared with healthy people carrying rs187238 CC genotype, people with CG genotype had a low risk of LC (Table 4, CG vs. CC: OR = 0.59, 95%CI = 0.38–0.91, *P* = 0.02). The genotype GG at187238 was more frequent in LC patients than CHB patients (2% vs. 0.8%) and logistic regression analyses showed a higher LC risk in CHB carriers with rs187238 GG genotype than individuals with CG or CC genotypes (GG vs. CC: OR = 6.25, 95%CI = 1.09–35.8; GG vs. CC+CG: OR = 5.91, 95%CI = 1.04–33.7). However, after controlling for gender, age, smoking and alcohol consumption, the result indicated no significant difference in rs187238 polymorphism between LC and health controls (Table 5). No relationship between rs1946518 polymorphism and LC susceptibility was detected (Tables 4 and 5).

**Table 4:** Association between IL-18 polymorphisms and LC and HCC risk

Model	LC vs. Controls		HCC vs. Controls		LC vs. CHB		HCC vs. CHB		HCC vs. LC	
Rs187238	OR (95%CI)	*P*	OR (95%CI)	*P*	OR (95%CI)	*P*	OR (95%CI)	*P*	OR (95%CI)	*P*
Allele										
C	1^ref^	0.10	1^ref^	0.95	1^ref^	0.11	1^ref^	0.98	1^ref^	0.12
G	0.73 (0.50-1.07)		0.99 (0.69-1.42)		1.37 (0.93-2.01)		1.004 (0.67-1.51)		1.36 (0.92-2.00)	
Codominant model										
CC	1^ref^		1^ref^		1^ref^		1^ref^		1^ref^	
CG	0.59 (0.38-0.91)	0.02	0.74 (0.48-1.13)	0.16	1.09 (0.70-1.70)	0.70	0.87 (0.55-1.37)	0.54	1.26 (0.80-2.00)	0.32
GG	2.26 (0.43-11.8)	0.54	4.40 (0.94-20.7)	0.04	4.81 (1.03-22.6)	0.03	2.28 (0.47-12.91)	0.47	1.94 (0.64-5.91)	0.23
Dominant model										
CC	1^ref^	0.04	1^ref^	0.02	1^ref^	0.32	1^ref^	0.75	1^ref^	0.19
CG+GG	0.64 (0.42-0.97)		0.85 (0.56-1.27)		1.24 (0.81-1.90)		0.93 (0.60-1.45)		1.33 (0.86-2.06)	
Recessive model										
CC+CG	1^ref^	0.44	1^ref^	0.03	1^ref^	0.03	1^ref^	0.44	1^ref^	0.26
GG	2.54 (0.49-13.2)		4.73 (1.01-22.1)		4.73 (1.01-22.1)		2.54 (0.49-13.2)		1.86 (0.61-5.63)	
Rs1946518	OR (95%CI)	P	OR (95%CI)	P	OR (95%CI)	P	OR (95%CI)	P	OR (95%CI)	P
Allele										
G	1^ref^	0.53	1^ref^	0.85	1^ref^	0.95	1^ref^	0.70	1^ref^	0.66
T	0.92 (0.72-1.18)		0.98 (0.76-1.25)		1.01 (0.79-1.29)		0.95 (0.74-1.22)		1.06 (0.82-1.36)	
Codominant model										
GG	1^ref^		1^ref^		1^ref^		1^ref^		1^ref^	
GT	0.85 (0.56-1.29)	0.44	0.91 (0.59-1.39)	0.66	0.80 (0.52-1.23)	0.31	0.75 (0.49-1.14)	0.17	0.92 (0.60-1.42)	0.72
TT	0.86 (0.53-1.40)	0.55	0.96 (0.58-1.56)	0.86	1.03 (0.62-1.70)	0.92	0.92 (0.56-1.52)	0.76	1.11 (0.68-1.81)	0.67
Dominant model										
GG	1^ref^	0.42	1^ref^	0.70	1^ref^	0.49	1^ref^	0.27	1^ref^	0.68
GT+TT	0.85 (0.57-1.26)		0.92 (0.62-1.38)		0.87 (0.58-1.30)		0.80 (0.54-1.19)		1.09 (0.73-1.61)	
Recessive model										
GG+GT	1^ref^	0.84	1^ref^	0.94	1^ref^	0.42	1^ref^	0.60	1^ref^	0.78
TT	0.96 (0.64-1.44)		1.02 (0.68-1.53)		1.19 (0.78-1.80)		1.12 (0.74-1.70)		1.06 (0.71-1.60)	

CHB, chronic hepatitis B; LC, liver cirrhosis; HCC, hepatocellular carcinoma; OR, odd ratio; 95%CI, 95% confidence interval.

**Table 5:** Association between IL-18 polymorphisms and LC and HCC risk adjusted by gender, age, smoking and drinking

Model	LC vs. Controls		HCC vs. Controls		LC vs. CHB		HCC vs. CHB		HCC vs. LC	
Rs187238	OR* (95%CI*)	P*	OR* (95%CI*)	P*	OR* (95%CI*)	P*	OR* (95%CI*)	P*	OR* (95%CI*)	P*
Allele										
C	1^ref^	0.51	1^ref^	0.82	1^ref^	0.04	1^ref^	0.78	1^ref^	0.23
G	0.85 (0.51-1.39)		1.05 (0.68-1.62)		1.60 (1.01-2.53)		1.06 (0.69-1.65)		1.29 (0.86-1.93)	
Codominant model										
CC	1^ref^		1^ref^		1^ref^		1^ref^		1^ref^	
CG	0.74 (0.43-1.28)	0.28	0.70 (0.41-1.18)	0.18	1.31 (0.76-2.27)	0.33	0.98 (0.60-1.60)	0.92	1.16 (0.70-1.90)	0.57
GG	2.25 (0.27-18.47)	0.45	6.96 (1.24-39.0)	0.03	6.25 (1.09-35.8)	0.04	2.09 (0.34-12.82)	0.42	2.38 (0.62-9.10)	0.20
Dominant model										
CC	1^ref^	0.37	1^ref^	0.52	1^ref^	0.12	1^ref^	0.94	1^ref^	0.35
CG+GG	0.78 (0.46-1.33)		0.85 (0.52-1.40)		1.52 (0.90-2.57)		1.02 (0.63-1.65)		1.26 (0.78-2.02)	
Recessive model										
CC+CG	1^ref^	0.41	1^ref^	0.02	1^ref^	0.046	1^ref^	0.42	1^ref^	0.22
GG	2.41 (0.29-19.82)		7.64 (1.37-42.5)		5.91 (1.04-33.7)		2.10 (0.34-12.85)		2.32 (0.61-8.81)	
Rs1946518	OR* (95%CI*)	P*	OR* (95%CI*)	P*	OR* (95%CI*)	P*	OR* (95%CI*)	P*	OR* (95%CI*)	P*
Allele										
G	1^ref^	0.73	1^ref^	0.56	1^ref^	0.37	1^ref^	0.72	1^ref^	0.78
T	1.06 (0.78-1.44)		0.91 (0.67-1.24)		0.87 (0.63-1.18)		0.95 (0.72-1.25)		1.04 (0.78-1.36)	
Codominant model										
GG	1^ref^		1^ref^		1^ref^		1^ref^		1^ref^	
GT	0.95 (0.55-1.62)	0.84	0.75 (0.44-1.28)	0.29	0.59 (0.35-1.01)	0.05	0.72 (0.45-1.15)	0.17	1.15 (0.72-1.84)	0.55
TT	1.12 (0.60-2.07)	0.72	0.84 (0.45-1.54)	0.57	0.76 (0.41-1.42)	0.39	0.92 (0.53-1.58)	0.76	1.07 (0.63-1.83)	0.80
Dominant model										
GG	1^ref^	0.99	1^ref^	0.32	1^ref^	0.09	1^ref^	0.27	1^ref^	0.60
GT+TT	1.002 (0.61-1.66)		0.78 (0.47-1.28)		0.64 (0.39-1.06)		0.78 (0.50-1.21)		1.12 (0.73-1.73)	
Recessive model										
GG+GT	1^ref^	0.57	1^ref^	0.97	1^ref^	0.79	1^ref^	0.58	1^ref^	0.93
TT	1.16 (0.70-1.93)		1.01 (0.61-1.67)		1.07 (0.64-1.80)		1.13 (0.72-1.78)		0.98 (0.63-1.54)	

CHB, chronic hepatitis B; LC, liver cirrhosis; HCC, hepatocellular carcinoma; OR, odd ratio; 95%CI, 95% confidence interval; OR*, OR values adjusted by gender, age, smoking and drinking; P*, *P* values adjusted by gender, age, smoking and drinking.

### Association between IL-18 polymorphisms and the risk of HCC

A significant higher risk of HCC was associated with rs187238 GG genotype, when compared with healthy controls with CC/CG genotypes (Table 4, GG vs. CC+CG: OR = 4.73, 95%CI = 1.01–22.1) and the similar trend was also observed after adjustment for gender, age, smoking and alcohol consumption (Table 5, GG vs. CC: OR = 6.96, 95%CI = 1.24–39.0; GG vs. CC+CG: OR = 7.64, 95%CI = 1.37–42.5). Unfortunately, whether compared with healthy group or CHB carriers, rs1946518 polymorphism did not have a significant influence on developing to HCC (Tables 4 and 5).

### Association between IL-18 polymorphisms and the risk from LC to HCC

Displayed in Table 5, neither of the two polymorphisms hampered or accelerated patients from LC progressing to HCC (Table 4). Adjusting for gender, age, smoking and drinking status did not have a meaning change (Table 5).

### Haplotype distributions of IL-18 polymorphisms in healthy controls, HBV, LC and HCC patients

We further performed the haplotype analyses to evaluate the haplotype frequencies of rs187238 and rs1946518 polymorphisms. Three haplotypes (G_rs187238_T_rs1946518_, C_rs187238_T_rs1946518_ and C_rs187238_G_rs1946518_) were analyzed. As shown in Table 6, the two polymorphisms had no interaction. However, after adjustment by gender, age, smoking and drinking, haplotype C_rs187238_T_rs1946518_ moderately decreased the risk of CHB carriers developing into HCC (Table 7, OR = 0.69, 95%CI = 0.50–0.96, P = 0.03).

**Table 6:** Analysis of IL-18 haplotype frequencies with the risk of LC and HCC

	HCC vs. Controls		HCC vs. LC		HCC vs. CHB		LC vs. Controls		LC vs. CHB	
Haplotypes	OR (95% CI)	*P*	OR (95% CI)	*P*	OR (95% CI)	*P*	OR (95% CI)	*P*	OR (95% CI)	*P*
GT	0.98 (0.68–1.40)	0.90	1.30 (0.90–1.87)	0.16	1.32 (0.91–1.92)	0.15	0.73 (0.49–1.07)	0.10	1.004 (0.67–1.50)	0.98
CT	0.99 (0.77–1.29)	0.96	0.94 (0.73–1.23)	0.67	0.88 (0.68–1.15)	0.36	1.05 (0.81–1.36)	0.71	0.94 (0.72–1.22)	0.63
CG	1.02 (0.79–1.30)	0.90	0.93 (0.73–1.19)	0.58	0.98 (0.77–1.27)	0.90	1.09 (0.85–1.39)	0.49	1.06 (0.82–1.36)	0.65

CHB, chronic hepatitis B; LC, liver cirrhosis; HCC, hepatocellular carcinoma; GT, G_rs187238_T_rs1946518_; CT, C_rs187238_T_rs1946518_; CG, C_rs187238_G_rs1946518_; OR, odd ratio; 95%CI, 95% confidence interval.

**Table 7:** Analysis of IL-18 haplotype frequencies with the risk of LC and HCC adjusted by gender, age, smoking and drinking

	HCC vs. Controls		HCC vs. LC		HCC vs. CHB		LC vs. Controls		LC vs. CHB	
Haplotypes	OR* (95% CI*)	P*	OR* (95% CI*)	P*	OR* (95% CI*)	P*	OR* (95% CI*)	P*	OR* (95% CI*)	P*
GT	1.01 (0.65–1.57)	0.96	1.28 (0.85–1.91)	0.24	1.55 (0.98–2.46)	0.06	0.85 (0.51–1.39)	0.51	1.06 (0.69–1.65)	0.78
CT	0.91 (0.66–1.25)	0.55	0.93 (0.70–1.24)	0.62	0.69 (0.50–0.96)	0.03	1.14 (0.83–1.56)	0.43	0.92 (0.69–1.23)	0.58
CG	1.09 (0.80–1.48)	0.59	0.95 (0.73–1.25)	0.74	1.14 (0.83–1.56)	0.43	0.95 (0.70–1.29)	0.73	1.05 (0.80–1.38)	0.72

CHB, chronic hepatitis B; LC, liver cirrhosis; HCC, hepatocellular carcinoma; GT, G_rs187238_T_rs1946518_; CT, C_rs187238_T_rs1946518_; CG, C_rs187238_G_rs1946518_; OR, odd ratio; 95%CI, 95% confidence interval.

## DISCUSSION

IL-18 promoter single-nucleotide polymorphisms (SNPs) were suggested to associate with different expression of IL-18 by causing differences in transcription factor binding [28]. Rs187238 polymorphism, which has a change from G to C changes the H4TF-1 nuclear factor binding site, while rs1946518 polymorphism disrupts a potential cAMP-responsive element binding protein binding site [16]. Furthermore, rs187238 polymorphism could affect significantly IL-18 transcriptional activity [29]. Rs1946518 polymorphism is highly linked with the pathogenesis of chronic C virus infection (HCV) and HCV-infected patients had higher levels of IL-18 that correlate with disease severity [30]. The two polymorphisms were also related to susceptibility to various diseases, such as tuberculosis, type 1 diabetes, and CHB [31–33].

Recently, the association between the two polymorphisms (rs187238 and rs1946518 polymorphisms) and HBV-related liver diseases were frequently researched. Lau HK et al. included 559 healthy controls and 342 HCC patients to investigate the relationship and finally found rs187238 GC and CC genotypes not only increased the risk of HCC, but also was responsible for vascular invasion and enhanced the prognosis in HBV-related HCC [34]. Rs1946518 polymorphism was associated with different outcomes of HBV infection and spontaneous clearance [35]. On the contrary, a retrospective research in Japan enrolled 204 HBV-infected cases from 1999 to 2003, of those 161 people developed to chronic progressive liver disease with 62 CHB patients, 52 LC patients, and 47 LCC patients [36]. A protect effect of -607AA genotype and -137C allele on the disease progression of HBV carriers was observed [36]. Rs187238C allele was related to HCC in codominant and dominant models and its C allele showed significantly lower promoter activities which may affect IL-18 production and further affect CHB progression [37]. However, several studies suggested reversed conclusions. Bao J et al. revealed rs187238 not rs1946518 polymorphism may be a protective factor against HCC [38]. The genotype and allele frequencies of rs1946518 polymorphism showed no significant differences between any two groups of healthy controls, CHB, and HCC groups [25]. A meta-analysis published in 2016, including eight studies about rs187238 polymorphism and seven studies about rs1946518 polymorphism, provided an evidence that the two polymorphisms had no influence on the risk of HCC in general or specifically of HBV-positive HCC [26].

Different from the previous studies, we enrolled 1000 people who were at different stages from health to HCC. The four groups did differ significantly in alcohol history and diabetes history. For LC patients carrying HBV, heavy alcohol consumption is a independent increased risk of HCC [39]. Lin CW et al. found in LC patients, the incidence of HCC was higher in patients with HBV infection and alcohol history than those with HBV infection or alcohol history alone [40]. Acetaldehyde, the ethanol metabolite, has carcinogenic characteristics by DNA binding [41]. Drinking-induced chronic oxidative stress and cytokine production could lead to LC and HCC development in the presence of chronic inflammation [42]. Alcohol could also promote HCC occurrence by deregulating the level of retinoic acid and S-adenosyl-L-methionine, and activing the Wnt/β-catenin pathway [43]. Diabetes could alter the hepatic function and the structure of the hepatocytes in the diabetic rats model, possibly by increasing hepatic oxidative stress, attenuating antioxidant capacity and further impairing liver [44, 45]. Our results indicated rs187238 GG genotype increased the risk of HCC in healthy population and the risk of LC in CHB carriers, and rs187238 CG genotype decreased the risk of LC in health population. Epidemiological studies had demonstrated several risk factors for liver cancer including tobacco exposure, alcohol consumption, diabetes mellitus, HBV and HCV infection [46]. Though there is a significant difference in diabetes history between healthy controls and cases, researchers had different opinions about the impact of rs187238 and rs1946518 polymorphisms on diabetes [32, 47]. So we controlled for age, gender, smoking and alcohol consumption to minimize any possible confounding of results by these factors and finally found health people with GG genotype still had a higher risk of HCC and CHB patients carrying GG genotype still had a higher risk of LC. But no association between rs1946518 polymorphism and HCC or LC risk was identified.

Our results should be interpreted with caution because of several limitations. Firstly, though we recruited 1000 samples in this study, the sample size of each group was relative small which may restrict its detail subgroup analysis by the clinical index. Secondly, considering we just controlled four factors (age, gender, smoking and alcohol consumption), other factors including environmental background, treatment protocols and living habits may cause some bias. Thirdly, all participants were all from Shaanxi Province, China, which may not stand for all the Chinese population. Lastly, the fundamental experiments should be further conducted to validate our results and explore the possible mechanism.

In conclusion, our research revealed that rs187238 GG genotype increased the risk of HCC in healthy population and the risk of LC in CHB carriers. However, rs1946518 polymorphism had no impact on CHB-related liver diseases. These results indicated that IL-18 rs187238 polymorphism may be involved in the progression of CHB-related diseases in Northwest Chinese population.

## MATERIALS AND METHODS

### Ethnics statement

This study was approved by the Ethics Committee of the Second Affiliated Hospital of Xi’an Jiaotong University (Xi’an, China). The research protocol was completed according to the approved guidelines.

### Study population

For the current analysis, we established a case-control study of 250 healthy controls, 250 CHB carriers, 250 LC patients and 250 HCC patients. HBV infection was detected by ELISA method that requiring positivity of serum HBsAg and serum negativity for anti-HCV. LC patients and HCC patients were confirmed by pathological diagnosed. All the subjects were from the Second Affiliated Hospital of Xi’an Jiaotong University, and Xijing Hospital of Fourth Military Medical University, Xi’an, Shaanxi Province, China. Every participant was interviewed in-person including questions on age, demographics, ethnicity, height and weight, smoking and drinking habits, and medical history. Blood samples were collected after interview.

### Genotyping assay

The samples were centrifugated and stored in −80°C freezers for long-term storage. The genomic DNA was extracted and concentrated by the method described in our previous studies [48, 49]. Two tag-SNPs (rs187238 and rs1946518) were selected in this study. SNP genotyping was performed by the Sequenom MassARRAY RS1000, and the primers were listed in Table 8. The data analyses were completed by Sequenom Type 4.0.

**Table 8:** Primers used in this study

SNP_ID	1st-PCRP	2nd-PCRP	UEP_SEQ
rs187238	ACGTTGGATGGCAGA GGATACGAGTACTTC	ACGTTGGATGACAGAGCC CCAACTTTTACG	GACCCAACTTTTACGGAAGAAAA
rs1946518	ACGTTGGATGCTCTCCCC AAGCTTACTTTC	ACGTTGGATGTATCAGAT GCAAGCCACACG	ACACGGATACCATCATTAGAATTTTAT

### Statistical analyses

All the statistical analyses were completed using the SPSS software package (version 20.0; SPSS Inc., Chicago, IL, USA). HWE was examined by comparing expected and observed frequencies using Alrlquin 3.1 program (L. Excoffier, CMPG, University of Bern, Switzerland). The genotype frequencies of observed values were compared with expected values obtained from HWE theory (p2+2pq+q2=1; p is the frequency of the wild-type allele and q is the frequency of the variant allele). Haplotype analysis was performed by PHASE v2.1 software. The calculation was performed by *χ2* test and the degree of freedom was 1 in the cases and controls. The significant difference in allele and genotype frequencies between cases and controls was determined by Pearson's *χ2* test. ORs and 95%CIs were calculated with and without adjustment for gender, age, smoking and drinking. We evaluated the risk in the dominant model (AA+ Aa *vs*. aa), the recessive model (aa *vs*. Aa+AA), and the allele model (a *vs*. A) respectively (A: the major allele, a: the minor allele). A two-sided *P*-value < 0.05 was considered statistically significant in all the tests.
